# PLEKHN1 promotes apoptosis by enhancing Bax-Bak hetro-oligomerization through interaction with Bid in human colon cancer

**DOI:** 10.1038/s41420-017-0006-5

**Published:** 2018-02-08

**Authors:** Sei Kuriyama, Tadahiro Tsuji, Tetsushi Sakuma, Takashi Yamamoto, Masamistu Tanaka

**Affiliations:** 1 Department of Molecular Medicine and Biochemistry, Faculty and Graduate School of Medicine, Akita University, Akita, Japan; 2 Department of Otorhinolaryngology-Head and Neck Surgery, Graduate School and Faculty of Medicine, Akita University, Akita, Japan; 3 Department of Mathematical and Life Sciences, Graduate School of Science, Hiroshima University, Higashihiroshima, Japan

## Abstract

The anti-apoptotic nature of cancer cells often impedes the effects of anti-cancer therapeutic agents. Multiple death signals influence mitochondria during apoptosis, and though many studies have attempted to elucidate these complicated pathways, Bax oligomerization, an important step in the process, remains controversial. Here we demonstrate that pleckstrin-homology N1 (PLEKHN1), also known as cardiolipin phosphatidic acid binding protein, plays pro-apoptotic roles during reactive oxygen species (ROS)–induced apoptosis. Human PLEKHN1 was expressed in several cancer cell lines of differing origin. Its expression was regulated by hypoxia, and it existed in the mitochondrial fraction. Genome editing of hPLEKHN1 in human colon cancer HT-29 cells revealed enhanced survival of knockout cells compared with that of parental cells in vitro and in vivo. Thapsigargin or hydrogen peroxide treatment activated multiple death signals including JNK, Bcl-2 family members, and caspases. PLEKHN1 was bound to Bid, a pro-apoptotic protein, and not to Bax, and PLEKHN1 could remove Bid from transient Bid–Bax complexes. Fluorescent time-lapse imaging revealed that PLEKHN1 aggregated with Bid during thapsigargin- or hydrogen peroxide-induced apoptosis prior to Bax aggregation. Inhibition of PLEKHN1 led to attenuation of Bax-Bak hetero-oligomerization and Bid translocation. The immunohistochemistry of cancer patient specimens showed that PLEKHN1 expression was absent from cancer region at the transition area of normal/cancer tissues. Collectively, the silencing of PLEKHN1 may be the key that cancer cells acquire the drug resistance.

## Introduction

Pleckstrin-homology N1 (PLEKHN1) was reported as cardiolipin phosphatidic acid binding protein^1^. It associates with microtubules and accumulates in RNA granules, which contain cytochrome-c mRNA^1^; however, its role in cancer has not yet been elucidated.

We were interested in the similarities between cancer cells and neural crest (NC) cells, which are similar to each other^2^. We searched NC-specific genes from the expression database in frog (XDB3.2, NIBB, JAPAN), and found that the frog homolog of PLEKHN1 was required for NC-development (unpublished data). This directed us to investigate the human PLEKHN1 homolog in cancer field.

In early stages of tumor development, cancer cells grow too fast, and move away from vein, hence cancer cells must survive low nutrition and lower oxygen partial pressure (hypoxia). Hypoxia triggers hypoxia-inducible factor, which alters gene expression and metabolic pathways^3,4^. Prolonged hypoxia causes oxidative stress and cellular cytotoxicity^5^.

The accumulation of reactive oxygen species (ROS) triggers apoptosis via inhibition of the anti-apoptotic factor, Bcl-2, or the activation of a proapoptotic factor, Bax, which induces apoptotic pore formation in the mitochondrial membrane and sequentially activates the caspase-3 pathway^6,7^. Bax is localized in the cytoplasm and translocates to the mitochondrial membrane^8^. Bid also translocates to the mitochondria and induces a conformational change in the N-terminal domain of Bax that coincides with cytochrome-c release^9^. Death receptor signaling then activates caspase-8, which digests Bid to a truncated form (tBid: p15)^10^, which enhances the oligomerization of Bak^11,12^ and Bax^13^. Bid or its BH3-peptide can enlarges the mitochondrial outer membrane (MOM) pore, and cardiolipin on the MOM is required for this pore formation^14^. Structural analyses revealed that a Bax–BH3 domain replaces Bax–Bid BH3-complexes, and this replacement nucleates Bax-oligomerization to induce apoptosis^15^.

It was recently demonstrated that Bax binds to the MOM as a monomer and then quickly self-assembles and active Bax does not exist as a unique oligomer but as several conjugates of dimer units^16^. Importantly, they suggested that cleaved Bid does not affect on Bax-assembly^16^, despite the translocation of cleaved Bid has been reported to lead mitochondrial dysfunction and apoptosome formation^17,18^. The double knock-out mice of Bax and Bak reduces apoptosis in response to certain death stimuli^19^. However, little is known about the mechanisms how Bax-Bak form complex, and how Bid involves in it.

We created a cell line, where hPLEKHN1-expression was depleted by genome editing using Platinum Gate TALEN^20^. Time-lapse imaging provided evidence that PLEKHN1 accumulates prior to Bax-aggregation, resulting in breakage of the MOM. Then, PLEKHN1 bound to Bid, but not to Bax, and could eluted Bid from Bid–Bax-complexes in vitro. These data suggest that PLEKHN1 swapped Bid for Bax from transient BH3-heterodimer. Taken together, we have identified a novel component of a well-known proapoptotic cascade.

## Results

### Genome structure and editing of PLEKHN1 gene

**Fig. 1:**
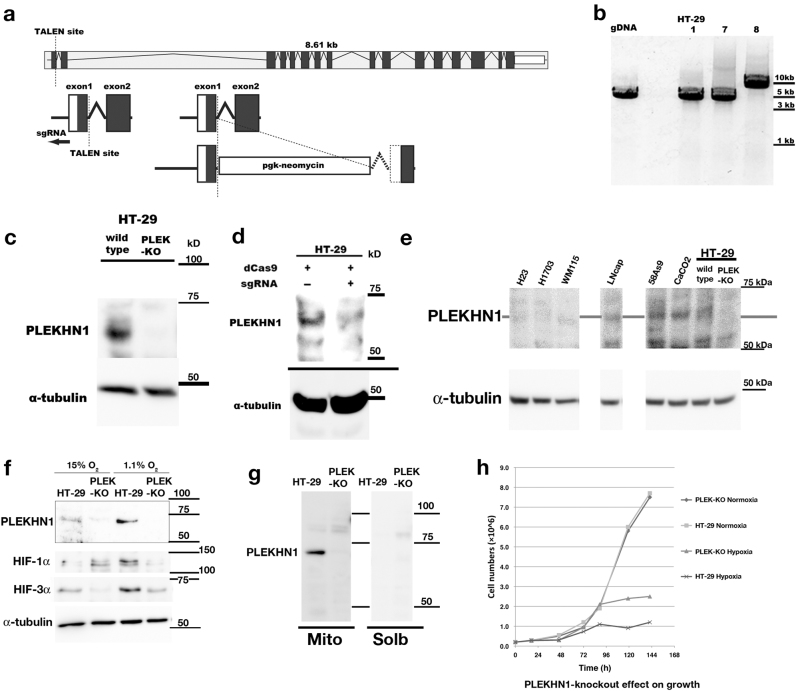
Expression profile of human PLEKHN1 and genome editing **a** Genomic structure and design of TALEN and sgRNA for CRISPR. Dark blocks indicate the exons. **b** Results of genomic DNA (gDNA) PCR. Clone 8 represents the PLEKHN1-KO cell line. **c** Western blot of HT-29 and PLEKHN1-KO cells using the anti-PLEKHN1 antibody that was created in our laboratory. **d** Comparison between pdCas9 (nuclease dead mutant)-expressing HT-29 and pdCas9 + pLXsgRNA-expressing HT-29 cells. The protein expression was diminished. **e** Western blot of several cancer cell lines. H23 and H1703 are lung adenocarcinoma. WM115 is melanoma. LNcap is prostate cancer cell line, and the faint expression was observed. 58As9 (scirrhous cancer), CaCO2 (colon cancer) and HT-29 (colorectal cancer) have 63 kDa bands, which is depleted in PLEK-KO cell. **f** Under hypoxic (1.1% O_2_) conditions, the level of PLEKHN1 was relatively high in HT-29 cells, while it was undetectable in PLEK-KO. The accumulation of HIF-1α, and HIF-3α indicates hypoxic condition. **g** Cell fraction of HT-29 or PLEK-KO cells; Mito fraction contains mitochondrial proteins, and Solb is the soluble fraction containing cytoplasmic proteins. **h** Cell proliferation in normoxic/hypoxic conditions. The number of PLEK-KO cells was increased under hypoxic conditions compared with that of parental HT-29 cells

The estimated full-length size of hPLEKHN1 is 63 kDa, and multiple alternatively spliced forms are predicted from genomic sequences. We made polyclonal antibody against PLEKHN1 because none of commercial products did work when we started this work, and used genome editing to obtain the evidence of gene expression. We created Transcription activator-like effector nuclease (TALEN) constructs for hPLEKHN1 exon1-2, and pgk-neomycin was inserted using homologous recombination (Fig. 1a). The single guide RNA (sgRNA) for clustered regulatory interspaced short palindromic repeat (CRISPR) targeted the predicted initiation site of PLEKHN1-transcription (Fig. 1a). We performed the genome editing in colon cancer cell line, HT-29, and the clone 8 had a large insertion in the genomic region (Fig. 1b), and the full-length hPLEKHN1 protein was abolished (Fig. 1c). Thus, we confirmed that our antibody recognized the full-length PLEKHN1 band. We also confirmed this result with the other knock-down cell line using nuclease-dead-Cas9 and sgRNA (Fig. 1d). These data confirmed that our antibody recognized the correct band, and we used these knockout and knock-down cell lines for functional analyses.

### Expression profile of human PLEKHN1

Simultaneously, we assessed several cell lines (Fig. 1e). In particular, digestive organ cancer cell lines, such as 58As9 (scirrhous cancer), CaCO2 and HT-29 (colon cancer) expressed the full-length PLEKHN1, and we could ignore the non-specific band by comparison of PLEKHN1-knockout-HT-29 (hereafter, PLEK-KO cells) and the parental HT-29 cells (Fig. 1e).

### Anaerobic metabolism induced expression of PLEKHN1

Rapid proliferation of cancer cells make themselves away from the vasculature, and result in low nutrition with hypoxic and acidic condition. We noticed that confluent or acidic culture medium increases PLEKHN1 expression (data not shown). Therefore, we tested if the expression of PLEKHN1 was induced under hypoxic conditions (Fig. 1f). PLEKHN1 was increased by hypoxia, which was indicated by the stabilization of hypoxia induced factor (HIF) proteins because *HIF1a* mRNA was not upregulated at this time (Supplementary Fig. 1a). We also performed a fraction analysis, which showed that PLEKHN1 is localized in mitochondrial fractions (Mito), and not in soluble fractions (Solb), in HT-29 cells (Fig. 1g), indicating that PLEKHN1 is somehow related to the respiration. Unfortunately, the concentrated endoplasmic reticulum (ER) or membrane fraction showed the multiple non-specific bands by PLEKHN1 antibody (data not shown).

Next, we assessed the effects of normoxic and hypoxic conditions on the cell growth rate. PLEK-KO cells acquired resistance against hypoxic conditions and the cell counts increased, whereas under normoxic conditions, the growth rate appeared to be unchanged (Fig. 1h). Therefore, the PLEK-KO phenotype may be involved in hypoxia-induced apoptosis or hypoxia-resistant cell growth.

### In vivo survival of PLEKHN1 KO cells

**Fig. 2:**
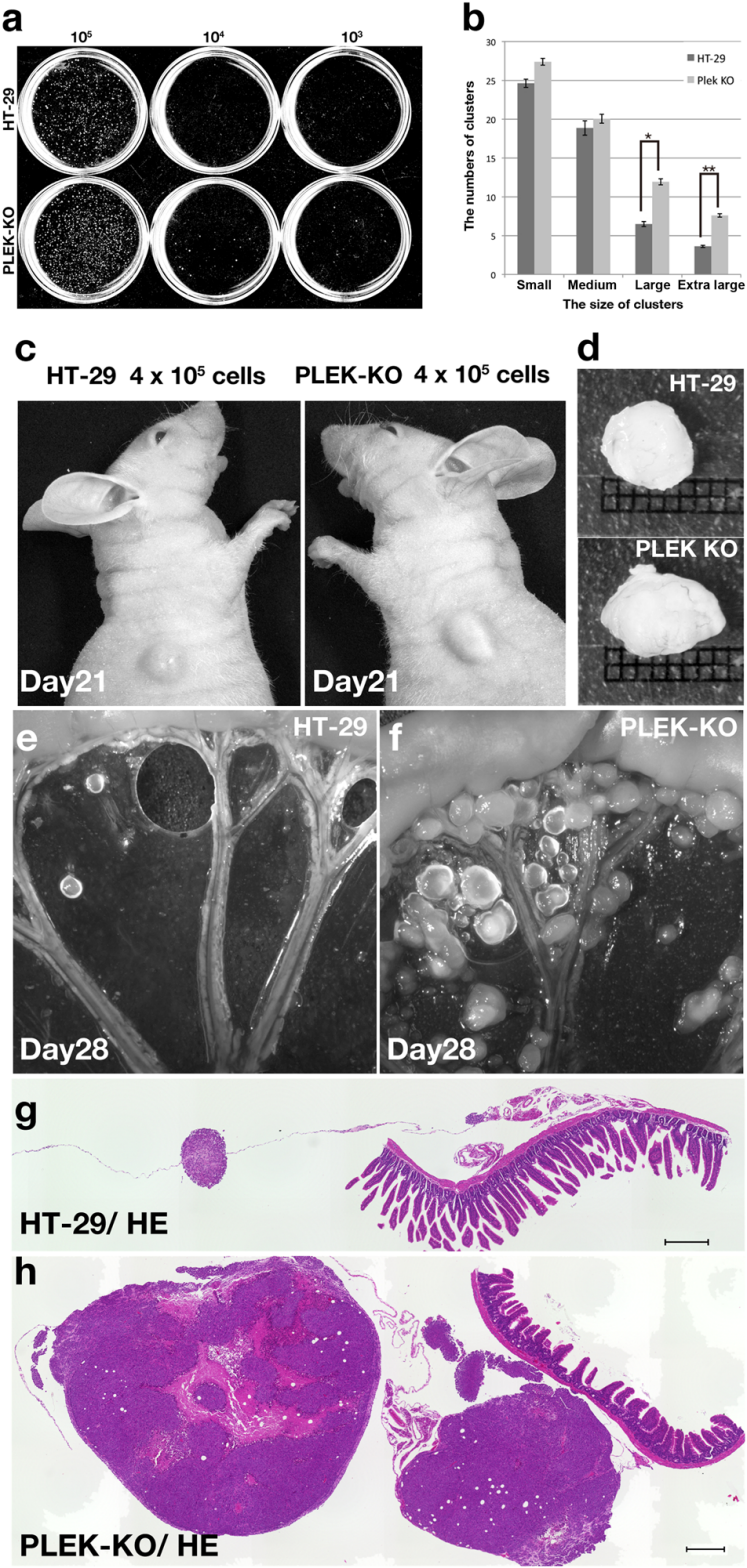
Gene targeting of PLEKHN1 increases the viability of HT-29 cells in vivo **a** Soft agar assay. White dots indicate cancer cell colonies. **b** The number of the cluster counts derived from the 10^3^ cell discs. The cluster counts were categorized by size: small cluster(less than 1000 µm^2^), medium cluster (from 1000 to 2780 µm^2^ (average size)) Large clusters (from average to 10,000 µm^2^) were significantly increased in PLEK-KO (*P = 0.025, double tail). The increase of the extra large (extraL) clusters (over 10000 µm^2^) in PLEK-KO was also siginificant (**P = 0.004, double tail). The error bars indicate the standard error. **c** Mice were subcutaneously injected with 4 × 10^5^ HT-29 or PLEK-KO cells. The images demonstrate the tumors 21 days following injection. The tumors formed by the PLEK-KO cells were larger than those formed by the HT-29 cells. **d** The tumors derived from PLEK-KO cells were larger than those of the HT-29 cells. The grid size is 1 mm^2^. (E, F) Mice were intraperitoneally injected with either HT-29 or PLEK-KO cells. Images depict mesenteric nodules at 28 days. **e** The HT-29 cells formed fewer mesenteric nodules compared with those by **f** PLEK-KO cells. **g** A hematoxylin-eosin(HE)-stained section of a mesenteric nodule. **h** The HE-stained section of mesenteric nodules of PLEK-KO cells. The diameter of the nodules was larger than that of those by the parental HT-29 cell. Scale bars indicate 300 µm

PLEKHN1-deficient cells may survive in large tumor, then, how about cell survival in unstable scaffold such as in body cavity? To guess PLEKHN1 function from knockout phenotype, we tested if the cells could colonize under anchorage-independent conditions with soft-agar assay (Fig. 2a). PLEK-KO cells formed larger colonies than those by HT-29 cells (Fig. 2a and b), implying that PLEK-KO might decrease anoikis. To test the effects of PLEKHN1-knockout on in vivo tumor outgrowth, we performed subcutaneous injections of cells (Fig. 2c). The cells were injected under the loose skin over the shoulder. In all cases, after 21 (*n* = 5) and 28 days (*n* = 9), PLEK-KO tumors grew larger than HT-29 tumors (Fig. 2c and d). As the malignant digestive organ cancer frequently spreads out to the mesentery, to test if PLEKHN1-knockout increase the malignancy, we performed intraperitoneal injections, and compared mesenteric nodules of HT-29 or PLEK-KO cells after four weeks (Fig. 2e, *n* = 5; Fig. 2f, *n* = 8). PLEK-KO nodules were increased compared with those of HT-29 cells (Fig. 2f). Both numbers and volumes of tumor nodules in PLEK-KO cells were increased (Fig. 2g and h). Dissemination of PLEK-KO cells in the injected mice was also frequently observed in the pancreas, spleen, and liver (data not shown). These data suggest that loss of PLEKHN1 in colon cancer may increase the risk of peritoneal dissemination.

### The resistance of anti-cancer therapeutic agents in PLEKHN1 KO cells

**Fig. 3:**
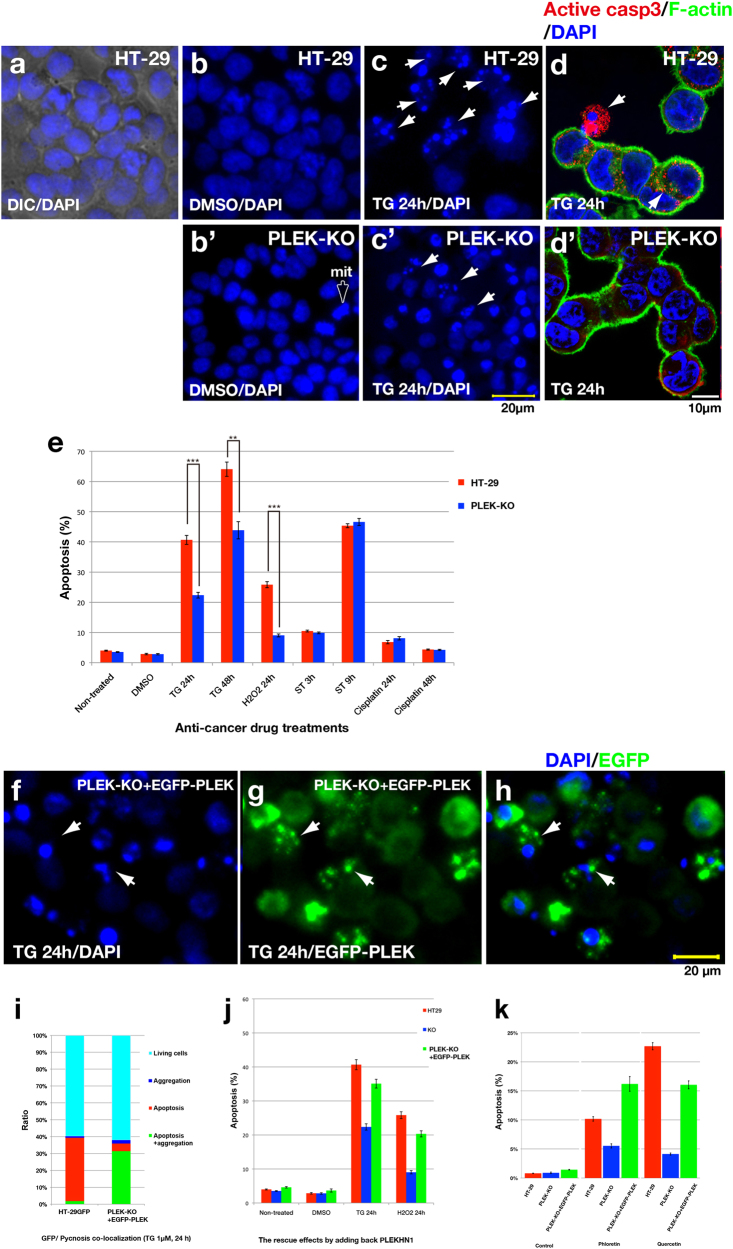
PLEK-KO cells reduce TG- or ROS-induced cell death **a** The image of DIC/DAPI staining. The merged images could be used to determine the proportion of apoptotic cells. **b** HT-29 cells in the presence of 0.1% DMSO with DAPI staining. **b**′ PLEK-KO of control medium. Arrow indicates cell during mitosis (mit) **c** HT-29 cells in the presence of 1 µM thapsigargin (TG)-treatment for 24 h. Arrowheads indicate fragmented nuclei. **c**′ PLEK-KO in 1 µM TG same as **c**. Less pycnotic cells were observed. **d** Immunofluorescence using active-caspase-3 antibody/Alexa546 rabbit IgG, FITC-phalloidin, and DAPI. Arrowheads indicate dead cells as measured by the accumulation of active-caspase-3 (depicted in red). **d**′ PLEK-KO cells as same as **d**. **e** The ratio of cell death after chemical treatments between HT-29 and PLEK-KO cells. The difference of the ratio of apoptosis in TG 24 h and H_2_O_2_ 24 h were significant (***P < 0.001), and that of TG 48 h were also significant (**P < 0.005). ST stands for staurosporin. The error bars indicate the standard error. **f** DAPI staining of PLEK-KO cells expressing EGFP-PLEKHN1 (EGFP-PLEK). **g** GFP fluorescence of PLEK-KO + EGFP-PLEK. Arrowheads indicate the aggregates of EGFP-PLEKHN1. **h** Apoptotic cell death co-localized with cells that contained EGFP-PLEK aggregates. **i** Ratio of the co-existence of EGFP-PLEK aggregations and cell death in the same cells. **j** The ratio of apoptotic cells following treatment with DMSO, TG, and H_2_O_2_. The apoptosis-resistant phenotype of the PLEK-KO was reversed with the addition of EGFP-PLEK. The error bars indicate the standard error. **k** The ratio of apoptotic cells with the treatments of plant flavonoid, Phloretin, and Quercetin. The color bars indicate same cells as **j**

To understand how PLEK-KO cells adapt to hypoxia, we analyzed apoptosis under various stressful conditions. Generally speaking, the colorectal cancer cells often have anti-cancer drug resistance. Thapsigargin (TG), an inhibitor of sarcoplasmic/endoplasmic reticulum Ca^2+^ dependent ATPase (SERCA), increases cytoplasmic Ca^2+^ levels, and induces apoptosis in HT-29 cells^21,22^. We stained the cells with DAPI, and counted the pycnotic cell death (Fig. 3a and b). The 1 µM TG treatment killed ~ 40% of HT-29 cells (Fig. 3c) within 24 h; however, its effect on PLEK-KO cells was weaker (Fig. 3c′ and e), while control medium had no effect on apoptosis to both cells (Fig. 3b, b′ and e). To confirm if cell death was apoptotic, we examined caspase-3 activation (Fig. 3d) whereas we could not find such typical staining from TG-treated PLEK-KO cells (Fig. 3d′). Active-caspase staining correlated with pycnotic cells, suggesting an apoptotic mechanism. HT-29 cells were gradually lysed by 100 µM H_2_O_2_ treatment over 24 h, and PLEK-KO reduced ROS-induced cell death (Fig. 3e). Cisplatin treatment did not induce enough apoptosis even in wide range of concentrations such as 0.5–120 µM (Fig. 3e, data not shown). To determine if the knock-down method had a similar effect on TG, we examined apoptosis in the cells used CRISPR/Cas system, and results indicated that knock-down of PLEKHN1 also decreased apoptosis (Supplementary Fig. 1b).

Next, to assess whether the cell death-resistance in the PLEK-KO cells was reversible with the addition of PLEKHN1, we established a sub-cell line of PLEK-KO expressing enhanced green fluorescent protein (EGFP)-fused to PLEKHN1 (PLEK-KO + EGFP-PLEK). In the absence of TG, GFP was observed in entire cytoplasmic region of PLEK-KO + EGFP-PLEK cells; however, fluorescence accumulated after 24 h of TG treatment and often coincided with apoptotic cell death (Fig. 3f, g, h, arrowheads). We measured aggregated GFP particles and fragmented nucleus, and demonstrated that aggregation of EGFP-PLEK was likely associated with apoptosis (Fig. 3i). Addition of EGFP-PLEK into PLEK-KO cells increased apoptosis to nearly the same level as observed for HT-29 cells (Fig. 3j). We also tested the anti-cancer drugs, which have been effective in HT-29 cells previously. Phloretin and Quercetin were both naturally existing plant flavonoid, causing apoptosis by different pathways^23,24^. PLEKHN1-knockout reduced the apoptosis, and add-back of EGFP-PLEK reversed knockout phenotypes (Fig. 3k). These data suggested that the resistance to cell death in the PLEK-KO cells was due to the depletion of PLEKHN1. Therefore, the western blot data and these results were mutually complemented (Fig. 1).

### TG drives JNK, CHOP, DR-5, Caspase-8, Bid, and PLEKHN1

Since thapsigargin is known as ER-stress inducer^25^, we tested ER-stress-induced apoptotic circuit: ER-stress activates JNK, CHOP, and DR-5 expession, then TRAIL activates caspase-8 via DR-5, and the activated caspase-8 cleaves Bid and enhances JNK, and cleaved Bid activates Bax and Bak to make the pore on MOM^18,26^. Since TG drives both intrinsic and extrinsic pathways of apoptosis, it is good to analyze unknown role of PLEKHN1 in apoptosis.

We found that TG-treatment increase the level of PLEKHN1, however, JNK phosphorylation were not changed in PLEK-KO (Supplementary Fig. 2a). Next, JNK inhibitor, SP600125 (SP) could reduce the apoptosis caused by TG and H_2_O_2_ (Supplementary Fig. 2b), therefore, we further followed ER-stress circuit. Next, the quantitative PCR of ER-stress marker, spliced XBP1, CHOP and DR-5 showed that the gene expressions were not changed by PLEKHN1-knockout (Supplementary Fig. 2c). H_2_O_2_-treatment in PLEK-KO did not alter the gene expressions too (Supplementary Fig. 2d), thus, further downstream may be involved in PLEKHN1-knockout phenotype. Finally, we tested the cleavage of caspase-8 and Bid. The protein cleavages were slightly altered, however, may not explain how the PLEKHN1-knockout reduced cell death (Supplementary Fig. 2e–h). Therefore, we next investigated if the localization of Bid or the other BH3 proteins directly inducing apoptosis via mitochondrial pathway.

### PLEKHN1 aggregates with Bid prior to Bax accumulation on mitochondria

The cytosolic Bax-monomer translocates to the MOM upon apoptotic stimulation^8,9^. To visualize this, we performed pulse-labeling of Bax. Tetramethylrhodamine-ligand (TMR) binds to HaloTag-Bax-fusion protein (TMR-Halo-Bax), which enables to visualize protein translocation^27^.

**Fig. 4:**
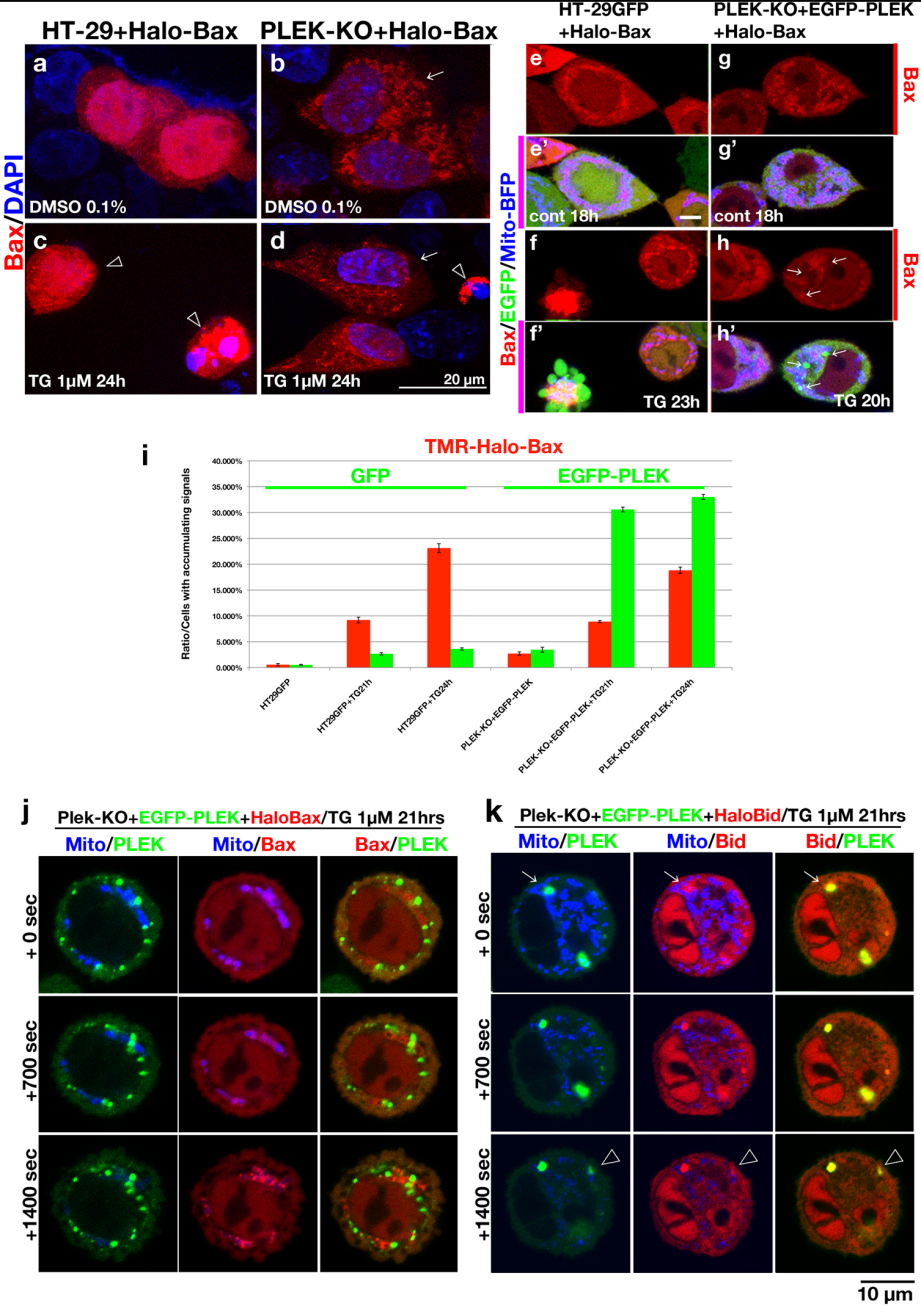
Subcellular localization of Bax or Bid is altered by PLEKHN1 The HaloTag-Bax construct was transfected into HT-29/PLEK-KO cells. Tetramethyl rhodamine (TMR)-labeled Halo ligand was applied to the samples, and fixed. **a** TMR-Bax was dispersed uniformly in the cytoplasmic region of the HT-29 control cells. **b** TMR-Bax was aggregated, but could be observed in the cytoplasmic region of the PLEK-KO control. **c** TMR-Bax accumulated in the mitochondria of HT-29 cells during TG treatment. **d** Most of the PLEK-KO cells escaped cell death, and the smaller clusters of TMR-Bax were observed in the cytoplasmic region (arrow). Some cells were rounded and TMR-Bax accumulated (arrowhead). **e**–**h** TMR-Bax and MitoBFP (mitochondrial marker) were transiently transfected into HT-29 + EGFP or PLEK-KO + EGFP-PLEK sub-cell lines. The upper columns depict TMR-Bax **e**–**h**, while the lower columns demonstrate Bax/EGFP/mitochondria (**e**′–**h**′). **e** HaloTag-TMR ligand labeling for 30 min, after 18 h of DMSO treatment. TMR-Bax and EGFP were observed uniformly in HT-29 cells. **f** After 23 h of TG treatment, The cell that TMR-Bax had accumulated in the mitochondria was collapsed (left). **g** TMR-Bax in PLEK-KO + EGFP-PLEK cells was observed uniformly. EGFP-PLEK was observed uniformly in the cytoplasm but not in the nucleus. **h** After 20 h of TG treatment, the arrows indicate that the aggregations consist of many EGFP-PLEK proteins. Bax had not yet aggregated. **i** Image analyses of TMR-Halo-Bax and EGFP particles. The accumulating signals were brighter than the basal intensity. The cells were counted with brighter particles from each of the same field of view. **j** Time-lapse imaging of TMR-labeled Bax in PLEK-KO + EGFP-PLEK cells, 21 h post labeling (0 s) to + 0, + 700, and + 1400 s. **k** Time-lapse imaging of TMR-labeled Bid in PLEK-KO + EGFP-PLEK cells, 21 h post labeling (0 s) to + 0, + 700, and + 1400 s. TMR-Bid co-localized with EGFP-PLEK (arrow). A newly formed particle was also double-positive (arrowhead)

First, we tested Bax-translocation with or without PLEKHN1 (Fig. 4a–d). TMR-Halo-Bax was uniformly distributed without any stimulation in HT-29 (Fig. 4a), and the strong accumulation was often coincided with fragmented nuclei after TG-treatment (Fig. 4c). Curiously, weak Bax-aggregation has been seen without TG-treatment in PLEK-KO (Fig. 4b), while futher aggregation did not occur after TG-treatment (Fig. 4d).

Second, we examined if PLEKHN1 localization was changed by cell death. HaloTag-Bax and mito-BFP were co-transfected into HT-29GFP (EGFP-expressing subcell line) or PLEK-KO + EGFP-PLEK cells. Under control conditions (+0.1% DMSO), both EGFP and EGFP-PLEK were uniformly distributed after 18 h (Fig. 4e and g). Both HT-29GFP and PLEK-KO + EGFP-PLEK cells were killed at similar ratio (Fig. 3i). EGFP remained uniformly distributed in HT-29GFP cells (Fig. 4f, TG for 23 h). However, EGFP-PLEK in PLEK-KO + EGFP-PLEK cells had already aggregated even when Bax and mito-BFP had not yet accumulated (Fig. 4h, TG for 20 h). We further quantified when the accumulation of TMR-Halo-Bax or EGFP-PLEK happened, we found that TMR-Halo-Bax was still increasing between 21 and 24 h of TG-treatment, while EGFP-PLEK had already been saturated at 21 h (Fig. 4i). Therefore, the order of accumulation of PLEKHN1 and Bax-translocation must be analyzed.

We confirmed the Bax-accumulation coincided with the leakage of cytochrome-c, while neither Bax-accumulation nor cytochrome-c distribution was observed in PLEK-KO cells (Supplementary Fig. 3a and b). It means that knockout of PLEKHN1 inhibits poring MOM. We also tested the localization of HaloTag-fusion protein of Bak (Halo-Bak), and TMR-Halo-Bak stayed on MOM regardless of PLEKHN1, and Bak-localization was not interfered by EGFP-PLEK (Supplementary Fig. 3c–e).

Finally, we performed time-lapse imaging, 21 h after TG-treatment, to determine the order of EGFP-PLEK-accumulation and Bax-translocation (Fig. 4j). Initially, TMR-Halo-Bax was evenly distributed and co-localized with mito-BFP while EGFP-PLEK has already accumulated (Fig. 4j, +0 s). Then, Bax accumulated on the MOM, finally mito-BFP disappeared (Fig. 4j, +1400 s). We repeated the experiment using HaloTag-Bid (Fig. 4k), and interestingly, EGFP-PLEK and TMR-Halo-Bid co-localized from + 0 s, and newly formed aggregates at the + 1400 s time-point did as well (Fig. 4k, +1400 s; arrowhead). This suggests that PLEKHN1 co-aggregates with Bid; Bax aggregates afterwards; and then the MOM disappears. The interaction between Bid and PLEKHN1 may cause Bax-translocation on the MOM, and changes its permeability.

### PLEKHN1 directly binds to Bid, not to Bax

**Fig. 5:**
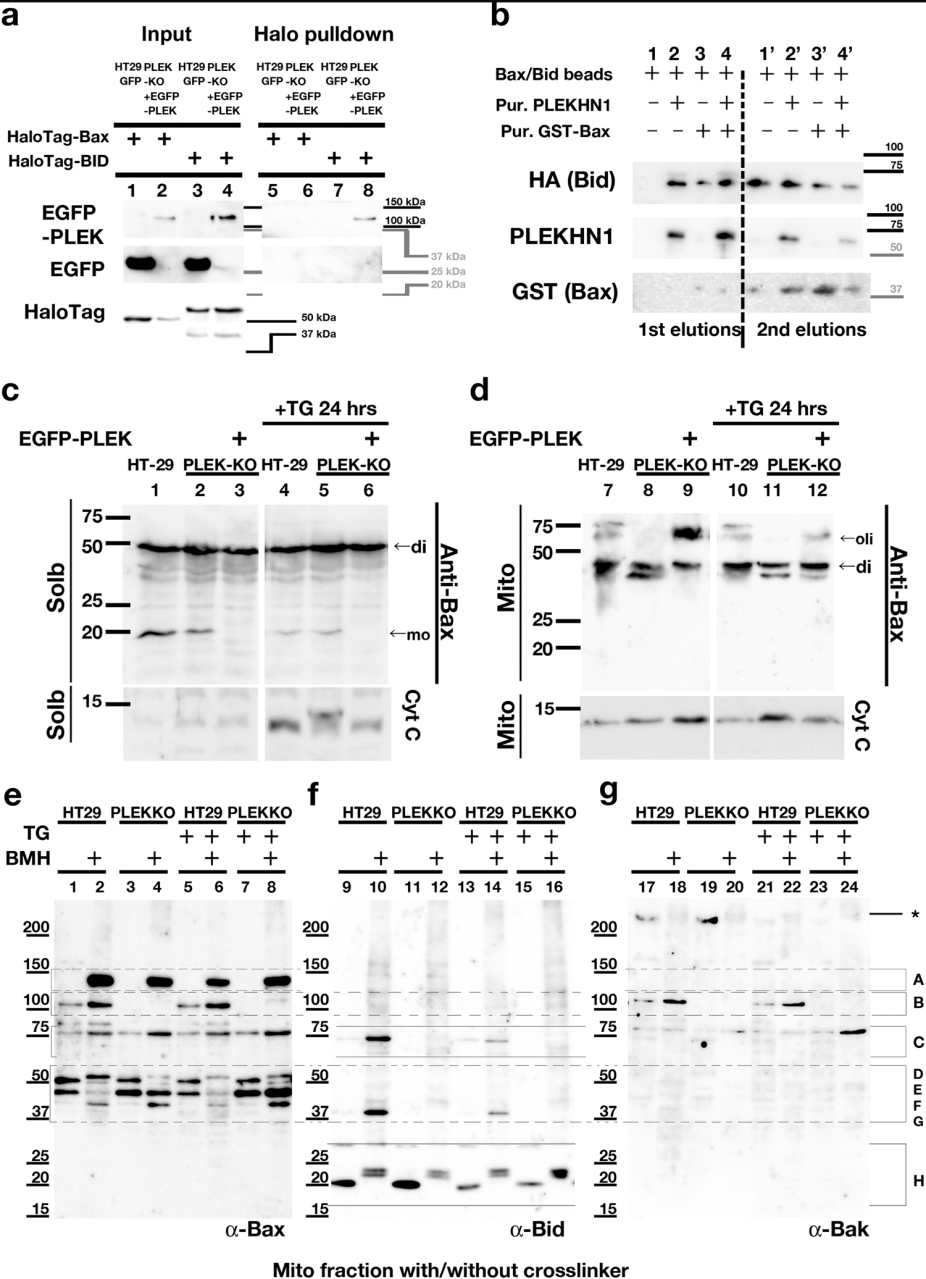
PLEKHN1 replaces Bid in Bax-Bid complexes to form a Bax–Bak complex. HaloTag constructs were transfected into HT-29GFP or PLEK-KO+EGFP-PLEK cells **a** Molecular interactions between PLEKHN1 and Bax or Bid. HaloTag protein complexes were pulled down and analyzed using a GFP antibody. Only HaloTag-Bid could pull down PLEKHN1 (lane 8). **b** GST-Bax and MBP-Bid-HA complexes were bound to GST-sepharose 4B.. The first elutions were obtained by adding purified PLEKHN1 protein. MBP-Bid-HA (Bid) was eluted without Bax (lane 2). The second elutions were obtained by adding the reduced glutathione, and indicated that similar amounts of proteins were bound to the columns. **c** The cell fraction analysis of HT-29, PLEK-KO, and PLEK-KO + EGFP-PLEK cells. Solb represents soluble proteins. TG treatment increased cytoplasmic translocation of cytochrome-c (lanes 4, 5, and 6). **d** Mito fraction represents mitochondrial proteins. In HT-29 cells, oligomerization of Bax had already occurred without TG-treatment (lanes 7, 10). The addition of PLEKHN1 to PLEK-KO cells restored the oligomerized Bax (lanes 9, 12). **e-g** To analyze the proximity of Bax or Bid, the mitochondrial fraction was obtained from the BMH-crosslinked protein samples, and loaded to 5–20% gradient gel with non-crosslinked protein samples. The different sizes of bands were labeled at the right end of Fig. 5g. **e** Immunoblot with anti-Bax antibody. The band A (~ 120 kDa) were observed every cross-linked samples. **f** Immunoblot with anti-Bid antibody. The major bands were seen in range C and G. **g** Immunoblot with anti-Bak antibody. The bands labeled with asterisk may be the undisolved large mitochondrial membrane fraction. The band B (~ 100 kDa) was overlapped with the band in 5e

To confirm the interaction between PLEKHN1 and Bid or Bax, we performed pulldown assays using HaloTag-Bax or HaloTag-Bid. Bid pulled down PLEKHN1, while Bax did not (Fig. 5a, lanes 6, 8). Then, we tested the competition for Bid of Bax and PLEKHN1 (see Supplementary Fig. 4). The purified PLEKHN1 alone could elute Bid without Bax, while Bax alone did not (Fig. 5b, lanes 1–3). Even when Bax were added with PLEKHN1, Bax and PLEKHN1 eluted nearly the same amount of Bid as PLEKHN1 alone (Fig. 5b, lane 4); thus, Bax did not interfere with PLEKHN1-Bid interaction. We confirmed that the equal amounts of Bid and Bax were bound on the column from the eluted protein by adding the reduced glutathione (Fig. 5b, 2nd elutions).

Next, we performed fraction-analyses of mitochondrial fraction (Mito) and soluble fraction (Solb) to determine how Bax and Bid were translocated. Bax was dimerized in Solb and Mito (Fig. 5c and d). In Mito of HT-29, bigger Bax-oligomers were seen (Fig. 5d, lane 7/10), however, in those of PLEK-KO, instead of Bax-oligomers were missing, lower size of Bax-dimer was increased (Fig. 5d, lane 8/11). Adding-back of EGFP-PLEK to PLEK-KO restored Bax-oligomers (Fig. 5d, lane 9/12), therefore, these data indicated that PLEKHN1 was required for Bax-oligomerization. Notably, cytochrome-c proteins were leaking to cytoplasmic region after TG-treatment (lane 4–6), and PLEK-KO cells still kept more cytochrome-c in mitochondrial fraction (Fig. 5d, lane 11). This indicated that the oxidative stress had already been elevated by the confluent culture, and TG pushed cancer cells slightly more to apoptosis.

Next, to determine the proximity of Bax or Bid, we performed fraction analyses using a bismaleimidohexane (BMH)-crosslinker, which force to make the complete oligomers when they are closely aligned (ref. 28). The crosslink revealed that most of the Bax in HT-29 cells were aligned as oligomers (Fig. 5e, band A-C), while Bax-dimers in PLEK-KO shifted to smaller from band D to E/F (Fig. 5e, lanes 2/4, 6/8), and Bax-band B (~ 100 kDa) was disappeared in PLEK-KO (Fig. 5e, lane 2/4, 6/8). This data indicated that PLEK-KO inhibited the Bax-alignment to form the oligomers from dimers. Bid-monomer and cross-linked proteins were uniformly seen (Fig. 5f, lane 9–16, band H). The crosslinked Bid appeared in HT-29 Mito-fractions (Fig. 5f, lane 10/14, band C and G), these bands were not visible in PLEK-KO (Fig. 5f, lane 12/16, band C and G). This implies that Bid actively works on the MOM in HT-29 cells, but not in PLEK-KO cells. Then, we also tested Bak expression, the other target of Bid. Interestingly, the band B was double-positive with Bax and Bak, and it was gone in PLEK-KO (Fig. 5g, lane 18/20). Another Bax-Bak hetero-oligomers (band C) were increased by TG (Fig. 5g, lane 24), while bands in B was disappeared (Fig. 5e, lane 8, Fig. 5g, lane 24). This data particularly explains why PLEKHN1-knockout reduced cell death and why TG could still kill PLEK-KO, because homo Bax-dependent pore remained and caused apoptosis even when the Bax-Bak-dependent pore reduced.

Taken all together, these data suggest that PLEKHN1 promotes Bax-Bak hetero-oligomerization likely via the removal of Bid from Bid–Bax complexes, or anchoring Bid to the MOM as a trigger of Bax-Bak hetero-oligomerization, or both.

### Immunohistochemistry of stomach cancer patient specimens

**Fig. 6:**
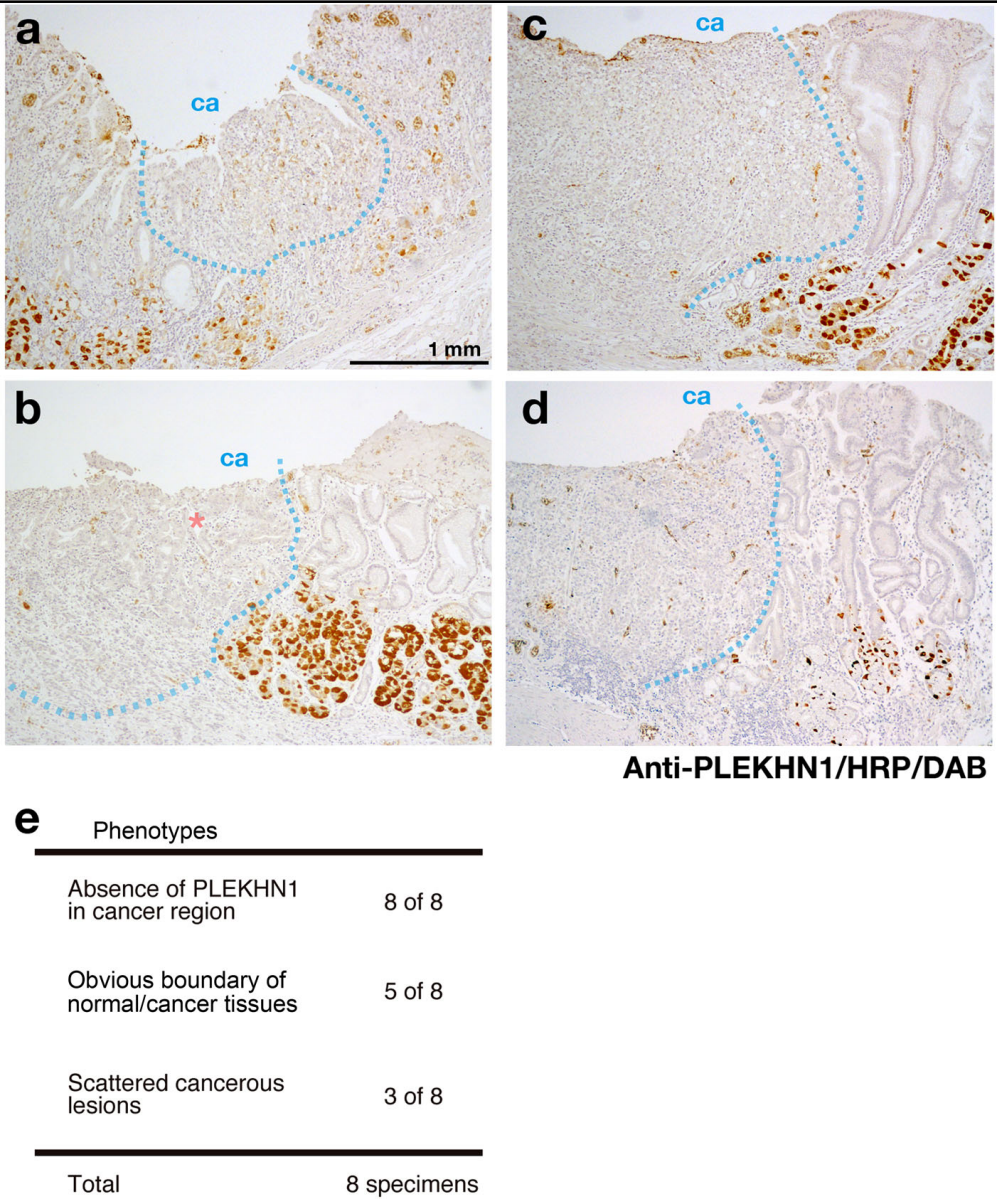
IHC of human stomach cancer specimens The sections of the stomach cancer patient specimens were stained by anti-PLEKHN1/HRP-rabbit polyclonal antibody/DAB. **a**–**d** The each section was from different patients. PLEKHN1 is expressed at wall cells of posterior stomach epithelium. The staining was not observed in cancer regions. The blue lines are visible border of cancerous tissue. “ca” indicates cancer side. **b** Asterisk indicates the isolated normal tubular structure. **e** Mini table of result summary

Loss-of-PLEKHN1 probably causes severe phenotypes in tumor, however, the endogenous colorectal expressions of PLEKHN1 were not high, this could be involved in the general drug resistance of colorectal cancer. Instead, the expressions of stomach were higher than those of colorectal tissues (data not shown). Therefore, to examine if the cancerous cells show the lower expression level of PLEKHN1 than that of the surrounding normal tissues, we performed IHC of randomly picked stomach cancer patient specimens with anti-PLEKHN1 antibody (Fig. 6). As far as we observed the samples at the transition area between normal and cancer tissues, PLEKHN1 was not observed in cancer cells, while normal stomach wall cells expressed PLEKHN1 (Fig. 6a–d). PLEKHN1 was absent in all specimen that we observed, however, the three of eight were too complicated to show the cancerous tissues with normal tissues (Fig. 6e).

## Discussion

Here we reported that PLEKHN1 acts as a novel member of pro-apoptotic proteins. Despite the expression of PLEKHN1 was induced by stresses, PLEKHN1-knockout increased the cancer cell survival in vivo, thus, PLEKHN1 is prepared for selection of the cells having some damages. In HT-29 cell line thapsigargin-treatment killed the cells by apoptosis, PLEK-KO cells showed the dull response to thapsigargin, suggesting that known apoptotic processes such as the cleavage of Bid and the translocation of Bax may happen nearby PLEKHN1 on mitochondrial membrane.

There are several models of Bax-oligomerization based on biochemical and biophysical analyses. First, to nucleate the core of Bax-oligomerization, Bax, Bid, and cardiolipin on the MOM are reportedly required^14^. Czabotar et al. described a model whereby monomeric Bax binds to the MOM, then an activator BH3-protein, such as tBid, transiently opens the “Latch”. The activator BH3-protein is displaced from the “Bax-Core,” and finally, each Bax-BH3-complex inserts its BH3-domain into the other Bax molecule’s BH3-groove to produce a Bax-dimer^15^. Here, we showed that the recombinant Bid–Bax complexes were dissociated by PLEKHN1 in solution (Fig. 5b), together our data with this model, PLEKHN1 accelerate to swap Bid over Bax to form Bax-dimer.

Stoichiometry-based quantification of Bax-assembly suggested the different model^16^. In their model, the Bax-monomer translocated to the MOM, quickly forming dimers, then these Bax-dimer-units formed various oligomers^16^. In our study, we also see most of Bax existed as the dimers, maybe band F was close to the predicted dimer size (42 kDa), thus most of Bax-dimers in HT-29 were somehow modified (Fig. 5c, d). Crosslinker analysis revealed that Bax formed ~ 120, 100, 75, and 50 oligomers/dimers, which may contain six, five, three to four and two BH3-proteins, respectively (Fig. 5e). Conversely, in the PLEK-KO cells, Bax-band D (50 kDa) turned to band F (42 kDa), which was not cross-reacted with anti-Bid or anti-Bak, thus, we guessed that Bax-band F might be an inactive form, however, further analyses of Bax-cleavage, phosphorylation, or cleaved Bid-binding etc. were required to clarify it. The crosslinking revealed that band B is likely Bax-Bak hetero-oligomer (Fig. 5e and g). Many combinations of Bax and Bak molecules are considerable, for example, the tetramer of Bax, two of the predicted dimers (42 kDa) and one Bak protein (23 kDa) could form the predicted pentamer of 107 kDa. Although further investigation is required for clarifying the function of Bax-Bak hetero-oligomers, it is only explanation that the disappearance of hetero-oligomers contributed to the reduction of apoptosis in PLEK-KO.

In PLEK-KO cells, Bid on the MOM was not detected (Fig. 5f, lanes 12/16), and it coincided with the disappearance of Bax–Bak hetero-oligomers (band B). Our pulse chase assay of Halo-Bax (Fig. 4j), Bid (Fig. 4k), and Bak (Supplementary Fig. 3c–e) showed that Bak stayed at MOM before the stimulation, Bid associated with PLEKHN1 from very early state of apoptosis, and Bax accumulated to MOM. However, PLEKHN1 did not bind to Bax, and not interfere Bak-localization (Fig. 5a and Supplementary 3e). Thus, PLEKHN1 may function as a scaffold for Bid. Maybe for the technical reason, it seems that the study of crystal structure of Bax–Bak heteromer have not yet been done, therefore, the other approaches using PLEK-KO cells to analyze Bax–Bak heteromer could be interesting.

Despite our mice in vivo experiments showed the possibility that loss-of PLEKHN1 from the primary lesion may cause the severe dissemination, we could not get such an appropriate specimens to follow that idea. Another idea is that PLEKHN1 level is lower in cancerous cells than that of normal cells. Although we could only gather a few samples, the patient specimens indicated clearly the absence of PLEKHN1 in cancerous tissues, which supported our in vitro and animal experiments and further suggest that lack of PLEKHN1 would be a key of acquirement of the drug-resistance.

## Material and methods

### Cell lines, culture, and the quantification of the cell proliferation

The lung adenocarcinoma cell line, H1703; the prostate cancer cell line, LNcap; the colon cancer cell line, CaCO_2_ were cultured in Dulbecco’s modified eagle medium (DMEM) with 1000 mg/mL glucose (Sigma-Aldrich, St. Louis, MO), 10% fetal bovine serum (FBS), and penicillin streptomycin (PS) (Sigma-Aldrich). The lung cancer cell line, H23; melanoma, WM115; the scirrhous stomach cancer cell line, 58As9, and the colon cancer cell line, HT-29 cells and derivatives were cultured in RPMI-1640 (Sigma-Aldrich) with 10% FBS and PS. To achieve the hypoxia condition, we adjusted the O_2_ level to 1.1% by filling N_2_ gas with 5% CO_2_ in multi-gas incubator (ASTEC Co Ltd, Fukuoka, JAPAN). For the measurements of cell proliferation, we prepared the multiple culture dishes in each 2 × 10^5^ cells/60 mm dish, and the cells were carefully trypsinized and counted with Bürker-Türk hemocytometer at the appropriate time points.

### Human PLEKHN1 antibodies

The fragment of the open reading frame of human PLEKHN1 cDNA (NM_032129) was amplified from a HaloTag-Kazusa cDNA clone DNA (FHC02940, Promega, Fitchburg, WV), and subcloned into a pGEX4 T vector with 6 × Histag sequences. Glutathione S-transferase (GST)-6 × His-PLEKHN1 N-terminal peptides (amino acids 6–143) were amplified in BL21(DE3)pLys cells (Thermo Fisher Scientific, Waltham, MA, USA), and purified using a Glutathione Sepharose 4B kit (GE Healthcare, Chicago). Further purification was accomplished using a His-tag column (Promega). Anti-hPLEKHN1 polyclonal antibodies were produced in rabbits, and immunoglobulins were purified using an affinity column (MBL, Nagoya, Japan).

### Antibodies

The antibodies used in this study are as follows: anti-active-caspase-3 rabbit polyclonal antibody (G7481, Promega), anti-Bax mouse monoclonal antibody (6A7, Sigma-Aldrich), anti-hBid rabbit polyclonal antibody (AF846, R&D, Minneapolis, MN), anti-Bak mouse monoclonal antibody (MAB8161, R&D), anti-HA tag antibody (HA.11/16B12, Covance Inc., Princeton, NJ), anti-Green fluorescent protein monoclonal antibody (mFX73, Wako, Osaka, JAPAN), anti-HIF1 alpha Rabbit polyclonal antibody (GTX127309, GeneTex, Irvine, CA) and anti-cytochrome-c Rabbit polyclonal antibody (GTX108585, GeneTex).

### Soft agar assay

As the basal agar solution, 0.5% agarose contained RPMI1640 + 10% FBS was prepared and warmed at 40 C° in hot water bath. The 6-well plate was coated with 2 mL basal agar. The cells were trypsinized, counted, and suspended in the appropriate concentration, then, 3 × 10^n^ cells (*n* = 3–5)/0.5 mL RPMI1640 was mixed with 1 mL of basal agar solution. This soft agar cell suspension (0.33%) was spread on the basal agar coated 6-well plate. After solidifying the agarose gels, RPMI1640 + 10% FBS was added on the gels, and the 6-well plate was cultured for 20 days. After fixation, the images of the colonies were obtained, and analyzed by Image J software. We ignored the thickness, and the colonies were quantified by the area. Small colonies are  < 1000 µm^2^, the average size of colonies was 2780 µm^2^, then 1000–2780 µm^2^ was middle, 2780–10,000 µm^2^ was large, and > 10,000 µm^2^ was called as extra-large. The counts of 10–15 fields were averaged, and compared between HT-29 and PLEK-KO.

### In vivo tumorigenesis

HT-29 or PLEK-KO cells were cultured in normal medium described above, and trypsinized, then, gently washed twice with RPMI1640 medium without FBS. The numbers of the cells were counted with Bürker–Türk counting chamber. The cells were further diluted to the appropriate concentrations with HBSS medium (Sigma-Aldrich). We injected 4 × 10^5^ cells/200 µL HBSS for subcutaneous injection, and 1 × 10^6^ cells/200 µL HBSS for intraperitoneal injection. The disposable syringe with 27 G needle was used for subcutaneous injection, and 29 G needle for intraperitoneal injection (Terumo corporation, Tokyo, Japan). The animal experiments were approved by Akita University’s ethical committee for the experimental animals.

### Anti-cancer drugs

Staurosporine (ST) was used at 500 nM (Wako Pure Chemical Industries (Wako), Osaka, Japan). Cisplatin, a DNA synthesis inhibitor, was used at 100 µM (Wako). H_2_O_2_ (100 mM) in distilled water was used at a 1:1000 dilution. Thapsigargin (TG), an inhibitor of sarcoplasmic/endoplasmic reticulum Ca^2+^ ATPase (SERCA), increases cytoplasmic leaking of Ca^2+^^21^. TG induces apoptosis via DR5-dependent death receptor pathway and mitochondria pathway as previously described^22^. Phloretin was used at 0.2 mM (Tokyo Chemical Industry (TCI) Co. Ltd, Tokyo, Japan). Quercetin hydrate was used at 120 µM (TCI).

### Genome editing

The expression vectors of platinum gate TALEN^20^ and target vectors (DT-A-Neo) were electroplated into HT-29 cells. The target sequences of TALEN are as follows: TALEN-exon1Left: 5′-GCCGACCTGTACGACT-3′, TALEN-exon1Right: 5′-AGCCACTGTGTCCCTC-3′. After 48 h, cells were trypsinized, spread onto several 10 cm dishes, and cultured in RPMI + 10% FBS containing 100 µg/mL G418 (Wako). The surviving colonies were collected, and the DNA of each colony was analyzed by polymerase chain reaction (PCR). For gene knock-down, the nuclease-dead form of Cas9 (pdCas9-humanized) was transfected into HT-29 cells. HT-29 + pdCas9 cells were selected by puromycin (1 µg/mL, Wako). The sgRNA sequences are: 5′-TCGTACAGGTCGGCAAGTCT(*ggg*)-3′. This sgRNA cDNA was cloned into pLX-sgRNA lentivirus vector. The pLX-sgRNA-transfected HT-29 + pdCas9 cells were selected by blasticidin S (10 µg/mL, Wako). The decrease of hPLEKHN1 mRNA was measured using quantitative reverse transcription PCR (RT-qPCR).

### Halotag-pull down

For HaloTag purification, cells were lysed using HaloTag purification buffer (50 mM Hepes, 1 mM DTT, 1 mM EDTA, 1 mM EGTA, 0.05% NP-40). Magne-HaloTag beads were blocked for 30 min using 0.1% BSA/HaloTag purification buffer. Then, the Magne-HaloTag beads were rinsed twice with HaloTag purification buffer, and mixed with 0.5 mg/mL protein lysates for 1 h at room temperature. The beads were gathered using a Magnetic stand (Promega) and rinsed with HaloTag purification buffer three times for 5 min at room temperature.

### Recombinant proteins and in vitro binding assay

The procedures were described in Supplementary Fig. 4a. Bax cDNA was subcloned into a pGEX4 T vector to make GST-Bax protein (GE healthcare). Bid-HA cDNA was subcloned into a pMAL-p2X vector to make Maltose-binding protein (MBP) fusion of Bid-HA (New England Biolabs, MA). The plasmids were transformed into BL21(DE3)pLys competent cells. The overexpressing bacterial pellets were lysed with *E. coli* lysis buffer (ECL: PBS, 0.1% TritonX-100, 50 mM EDTA, protease inhibitor cocktail, 1 mM PMSF). GST-Bax protein was obtained using a Glutathione sepharose 4B kit (GE healthcare), and MBP-Bid-HA was obtained using amylose resin (New England Biolabs). The column-bound Bax or Bid was washed three times with ECL buffer, and the GST-Bax proteins were eluted using 30 mM reduced glutathione-containing lysis buffer. MBP-Bid-HA complexes were eluted by maltose-containing ECL buffer. The proteins were dialyzed with PBS in dialysis cassettes (Slide-A-Lyzer G2 3 K, Thermo Fisher Scientific). The proteins in dialysis cassettes were directly concentrated with Spectra/Gel absorbent (Spectrum Laboratories, Inc. Rancho Dominguez). To avoid denature of the structure of Bax protein by higher concentration of Triton X-100, the concentrated proteins were quantified, and diluted in octyl glycoside buffer (OG buffer: 10 mM TrisHCl (pH 7.4), 150 mM NaCl, 0.1% octyl glycoside (Sigma-Aldrich). The HaloTag-PLEKHN1 construct was transfected into 293gp cells, and the protein was purified using HaloTag beads. TEV protease digestion of the HaloTag and PLEKHN1 junction eluted PLEKHN1. The recombinant Bax and Bid proteins were applied to the Glutathione sepharose 4B beads. The column was washed by OG buffer once, and the protein was eluted by PLEKHN1 protein (and Bax) containing or control OG buffer. To see if the similar amounts of proteins were bound to each GST column, 30 mM reduced glutathione containing OG buffer were added into column after the first elution (Fig. 5b, second elutions).

### Cell lysis and fraction analysis

Cells were treated with thapsigargin for 24 h. Prior to cell lysis, the adherent cells were washed with cell rinse buffer (20 mM TrisHCl (pH 7.4), 1.38 mM NaCl) to remove FBS, and treated with or without the bismaleimidohexane (BMH) crosslinker, which has 13 Å spacer, at 5 mM (Thermo Fisher Scientific) for 15 min at room temperature. To prevent non-specific crosslinking between extracellular and cytoplasmic proteins, the crosslinker reagents were applied to the cells and washed before cell lysis. Then, cells were lysed in hypotonic buffer A^28^ (250 mM sucrose, 20 mM Hepes (pH7.5), 10 mM KCl, 1.5 mM MgCl_2_, 1 mM EDTA, 1 mM EGTA, and proteinase inhibitors) with 21 G needle and syringe. Cell lysates were centrifuged at 750 × *g* for 10 min at 4 °C to remove large cell debris. The supernatants were centrifuged at 10,000 × *g* for 10 min at 4 °C. The pellets contained the mitochondrial proteins (Mito). These supernatants were centrifuged at 100,000 × *g* for 1 h at 4 °C. The pellets contained the ER and microsomes. The supernatant contained cytosolic proteins (Solb). Each fraction was concentrated using a 0.5 mL Microcon filter (Merck Millipore, Darmstadt, Germany).

### Immune fluorescence

The cells were fixed in 4% paraformaldehyde solution in PBS for 15 min, and permeabilized for 30 min in PBS + 0.01% Triton X-100 (PBST). The specimens were blocked with 5% donkey serum in PBST, incubated with antibody solution for 3 h at room temperature, and washed five times by PBS + 0.1% Tween-20 (Ptw). The secondary antibody used was Alexa546 anti-mouse (or rabbit) IgG (Thermo Fisher Scientific). The application of the secondary antibody was the same as that for the primary antibody, and the specimens were mounted using the Mowiol 4–88 antifade reagent, DABCO (Sigma-Aldrich).

### Live cell imaging, confocal microscopy and image quantification

HT-29 and its derivative cells were seeded four days prior to time-lapse-recording, and transfected HaloTag labeled Bax was added at day two. Then, cells were treated with 1 µM TG at day three. To monitor the state of mitochondria, mito-BFP was co-transfected. Mito-BFP is fusion protein of tagBFP and Cox-4’s mitochondrial localization signal peptides^29^. Mito-BFP construct was gift from Gia Voeltz (Addgene plasmid #49151). After 44 h of DNA transfection and 20 h of TG treatment, the transfected cells were stained with membrane permeable HaloTag tetramethyl rhodamine (TMR) ligand (Promega) for 30 min, carefully washed twice with PBS and once with RPMI medium, then the cells were seeded back into RPMI + 10% FBS containing 1 µM TG. For the quantitative image analyses of Hoechst33342-stained (or DAPI-staining for fixed cells) pycnotic/normal cells, TMR-Bax or GFP/EGFP-PLEK accumulation/uniform expression, we obtained each two pictures from 10 to 15 fields. To distinguish the accumulating particles from the diffused fluorescence, the one photograph was taken at the minimum exposure time that the strong signal appeared, and the other was taken at the maximum exposure for counting total cells.

The time-lapse images were obtained every 2 min for 60–120 min, and Z-stack images were also obtained using an LSM 780 confocal microscope system (Zeiss, Oberkochen, Germany). The Z-stack/time-lapse images were processed using IMARIS software (Bitplane, Zurich, Switzerland) or Zen software (Zeiss).

### Specimens from cancer patients and immunohistochemistry

Gastric adenocarcinoma specimens were obtained from eight patients who had undergone resection of primary gastric tumors. None of the patients had undergone preoperative radiation or chemotherapy. The study was approved by the ethical review board of Akita University, and all samples were collected from the surgical pathology files at Akita University Hospital, between 2008 and 2015 and tissues were obtained with the informed consent of the patients. The staining and coloring procedures are described in elsewhere of the previous paper^30^. For activation of antigen, we used pH6 treatment (Dako, Santa Clara, CA, USA).

## Electronic supplementary material

Supplemental figure legends

Figure S1

Figure S2

Figure S3

Figure S4
