# Supplementary material for: PLEKHN1 promotes apoptosis by enhancing Bax-Bak hetro-oligomerization through interaction with Bid in human colon cancer
Source: Cell Death Discov. 2018 Feb 8;4:11. doi: 10.1038/s41420-017-0006-5 (PMC5841295; doi:10.1038/s41420-017-0006-5)
Supplement: Supplementary file 1 — Supplemental figure legends [file 41420_2017_6_MOESM1_ESM.docx]

**Supplementary method**

**CRISPR/Cas9 system**

The nuclease-dead Cas9 (dCas9) was transfected to HT-29 by pdCas9 retrovius vector. The clone was selected by puromycin-resistant gene, and subcloned as HT-29+pdCas9. Furthermore, pLx lentivirus vector expressing single guide RNA (sgRNA) for human PLEKHN1 was also transfected to HT-29+pdCas9 subcell line. The clone was selected by blastocysin-resistant gene. The sub-cell line was named as HT-29+pdCas9+Plek sgRNA.

**Antibodies used in supplemental figures**

Anti-caspase-8 rabbit polyclonal antibody (1C12, Cell Signaling & Technology (CST), Danvers, MA), anti-phosphorylated SAPK/JNK (Thr183/Tyr185) rabbit monoclonal antibody (98F2, CST), anti-JNK1/3 rabbit polyclonal antibody (C-17/sc-747, Santa Cruz Biotechnology, Dallas, TX),

**Supplementary figure legends**

**Figure S1 Gene regulation in hypoxia and gene knock-down by CRISPR**

. a) Transcriptional regulation under hypoxic culture condition. HT-29 and PLEK-KO cells were cultured in normoxia (15 %O_2_) or hypoxia (1.1 %O_2_). After 24 hours incubation in hypoxic condition, HIF-1a mRNA expression in HT-29 or PLEK-KO was downregulated. Stress sensitive mRNA, CHOP was upregulated. Death receptor-5 had not yet been upregulated. (b) TG-induced cell death in HT-29 PLEKHN1 knockdown cells by CRISPR technology. Control (dCas9 only) and targeted (dCas9+sgRNA) were treated with 0.1 % DMSO or 1 µM Thapsigargin containing 0.1 % DMSO for 20 hours. And their nucleus were stained with Hoechst33342, and the normal and the fragmented nucleus were counted under the fluorescent microscope. Graph showed the ratio of apoptotic celle. HT-29+dCas9+Plek sgRNA cells survived better than dCas9 expressing HT-29.

**Figure S2 TG drives JNK, CHOP, DR-5, caspase-8, Bid and PLEKHN1**

(a) The phosphorylation of JNK1 (denoted by p-JNK1) in HT-29 and PLEK-KO cells during treatment with 1 µM TG. After TG treatment PLEKHN1 expression was upregulated. JNK phosphorylation increased 18 hours since treatment with 1 µM TG. The graph indicates the averaged levels of phosphorylated JNK bands. PLEKHN1 knockout did not interfere the upstream of JNK. (b) The effect of SP600125 (SP), a JNK-target inhibitor, on Ca^2+^ pathway-induced apoptosis. The apoptosis induced by 1 µM TG and H_2_O_2_ were blocked by SP in the HT-29 cells. This result indicate that PLEKHN1 is involved in JNK downstream. ((c, d) Quantitative RT-PCR analyses of TG/H_2_O_2_-treated samples in HT-29/PLEK-KO. (c) RT-qPCR analyses of cells with thapsigargin treatment (1 µM) for 12-24 hours. The peak of TG-effects on transcription was observed within 12-24 hours. (c) RT-qPCR analyses of cells with H_2_O_2_-treatment for 12-24 hours. The transcriptions were not affected. (e) Western blot of cell lysate of TG/TG+SP treated HT-29 or PLEK-KO cells for 24-36 hours. (f) Full-length caspase-8 amounts. One-way ANOVA (F=2.31, p=7.111 E-05<0.01) (g) The amounts of cleaved caspase-8. One-way ANOVA (F=2.31, p=0.00054 <0.01) (h) The amounts of full-length Bid. One-way ANOVA (F=2.31, p=0.0063 <0.01). All error bars in this figure indicate the standard error.

**Figure S3 The endogenous Bax aggregations and Bak localization**

H2B-GFP and Mito-BFP were transfected in HT-29 or PLEK-KO, and the cells were treated with TG 1µM, then the apoptosis was monitored by Nuclei-GFP. The cells were fixed and stained with anti-Bax and anti-cytochrome-c antibodies. The obtained fluorescent images were analyzed, and the pseudo-colors were applied to each channel. Bax is red, mitochondria is blue, and cytochrome-c (cytC) is green. (a) The endogenous Bax and cytC in HT-29 or PLEK-KO cells were immunostained. TG-treatment increase the accumulation of the endogenous Bax and diffusion of cytC in cytoplasmic regions. However, cytC diffusion was not obvious in PLEK-KO cells. (b) The overlap between Bax and cytC was analyzed by ImageJ software. The white color indicates the overlap between Bax and cytC. This result suggested that TG-treatment increased the permeability of mitochondrial membrane in HT-29, not in PLEK-KO. (c-e) HaloTag-Bak and mitoBFP expression vectors were transiently transfected to HT-29GFP and PLEK-KO+EGFP-PLEK cell lines. HaloTag fusion proteins were labeled by tetramethylrhodamine (TMR)-conjugated HaloTag ligand. (c) Without any stimulation, TMR-labeled Bak has already been on mitochondria. (d) Bak localization in PLEK-KO+EGFP-PLEK with control medium. TMR-Halo-Bak did not change its localization. (e) TG 1 µM after 24 hours, PLEK-KO+EGFP-PLEK cells started to die, however, EGFP-PLEK and TMR-Halo-Bak did not overlap each other.

**Figure S4 Competition for Bid of Bax and PLEKHN1**

(a) Schematic diagram of the experiment shown in Figure 5b. GST-Bax and MBP-Bid-HA proteins were induced in bacteria, the lysed recombinant proteins were purified by GST sepharose 4B and Amylose resin, respectively. Each purified proteins were eluted, and dialyzed, then the recombinant Bax and Bid proteins were diluted into octyl glycoside (OG) buffer, and applied to New GST sepharose 4B column. After making Bax-Bid complex on the column, the column was washed several times, and devided into small portions until use. PLEKHN1 protein was also purified, concentrated, and diluted in OG buffer. As the first elution, the purified PLEKHN1-containing (or without) OG buffer was applied to the aliquot of Bax-Bid column. After the overnight incubation at 4 Cº, the column was separated by the centrifuge, and the supernatant was collected as 1^st^ elute (Figure 5b, lane 1-4). PLEKHN1 bound to Bid and remove Bid from GST-Bax (right upper box). The columns were further washed, and the reduced glutathione containg OG buffer was applied, and the eluted proteins were collected as 2^nd^ elute (Figure 5b, lane 1’-4’). Whole complexes were removed from GST-sepharose (right lower box). (b) CBB staining of SDS-PAGE gel analyzed purified recombinant proteins. (c) GST-Bax and MBP-Bid-HA purified proteins (left picture). GST-Bax alone and GST-Bax with MBP-Bid-HA were applied to new GST-sepharose, and washed several times, and eluted by the reduced glutathione. Even when MBP-Bid-HA was co-eluted, equal amounts of GST-Bax were eluted from each column, therefore, Bid binds to Bax not to column. The aliquots used in Figrue 5b are same aliquot as Figure S4c, lane 4.
