# Supplementary figures and images for: PLEKHN1 promotes apoptosis by enhancing Bax-Bak hetro-oligomerization through interaction with Bid in human colon cancer
Source: Cell Death Discov. 2018 Feb 8;4:11. doi: 10.1038/s41420-017-0006-5 (PMC5841295; doi:10.1038/s41420-017-0006-5)

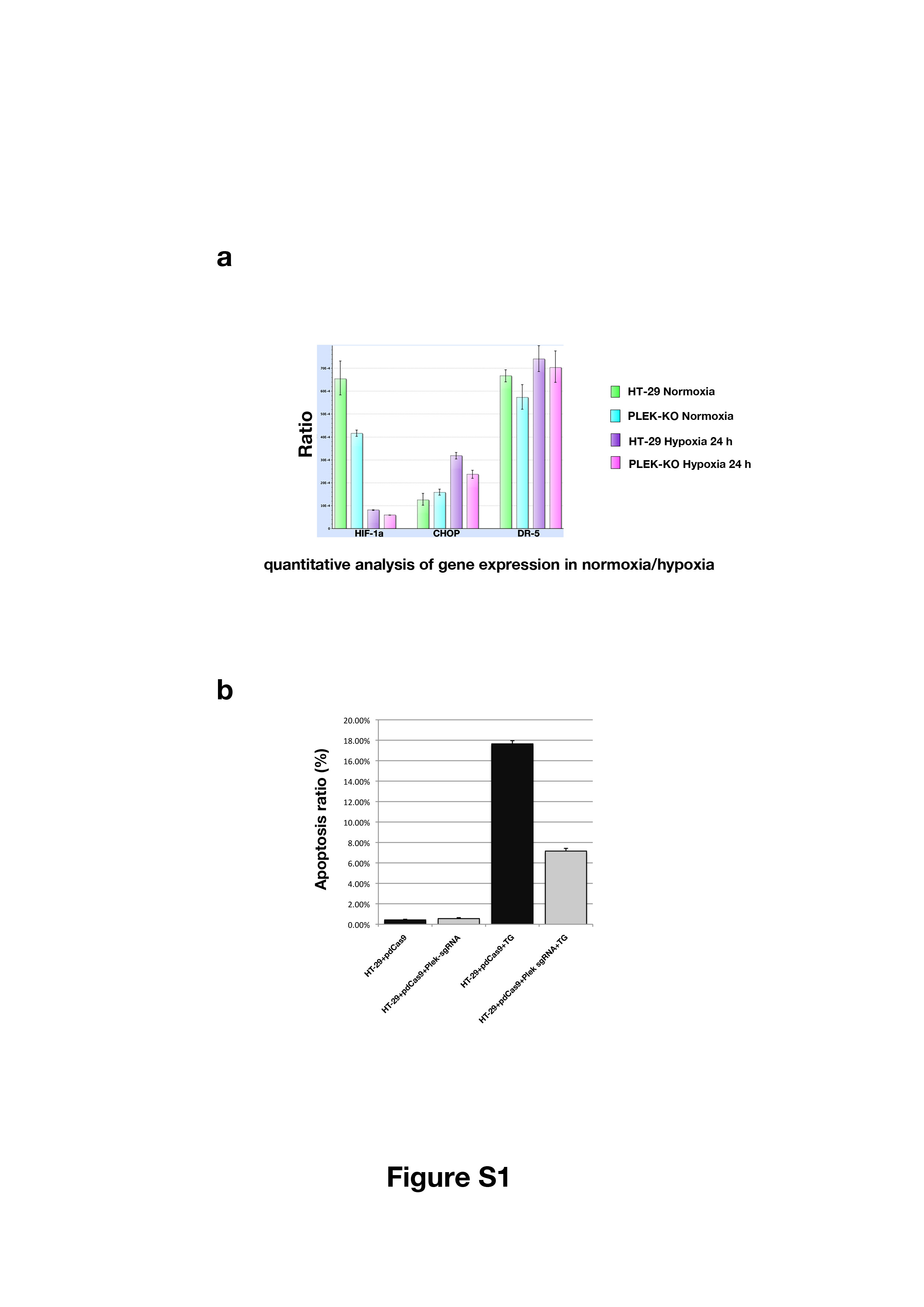

Supplement: Supplementary file 2 — Figure S1 [file 41420_2017_6_MOESM2_ESM.jpg]

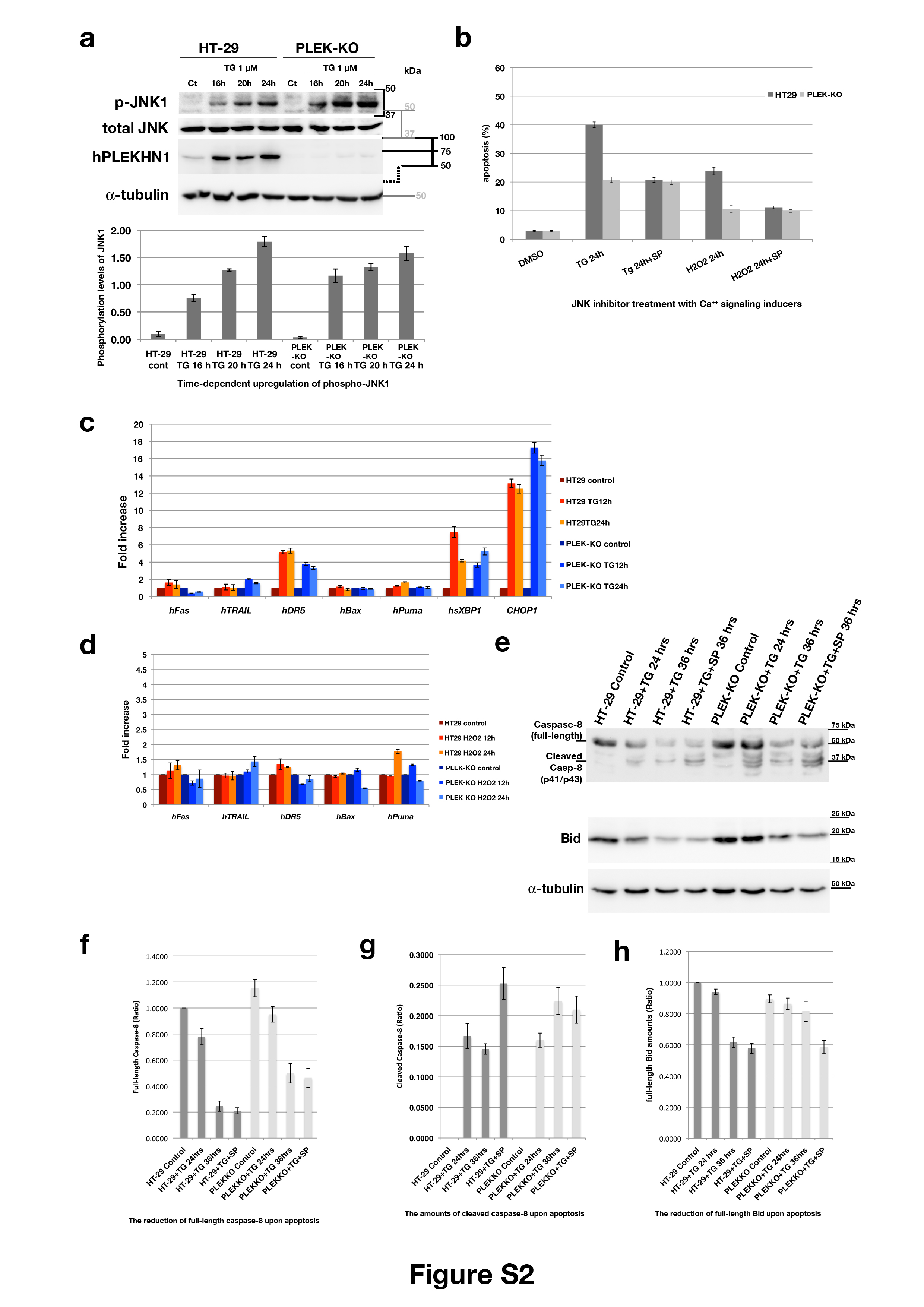

Supplement: Supplementary file 3 — Figure S2 [file 41420_2017_6_MOESM3_ESM.jpg]

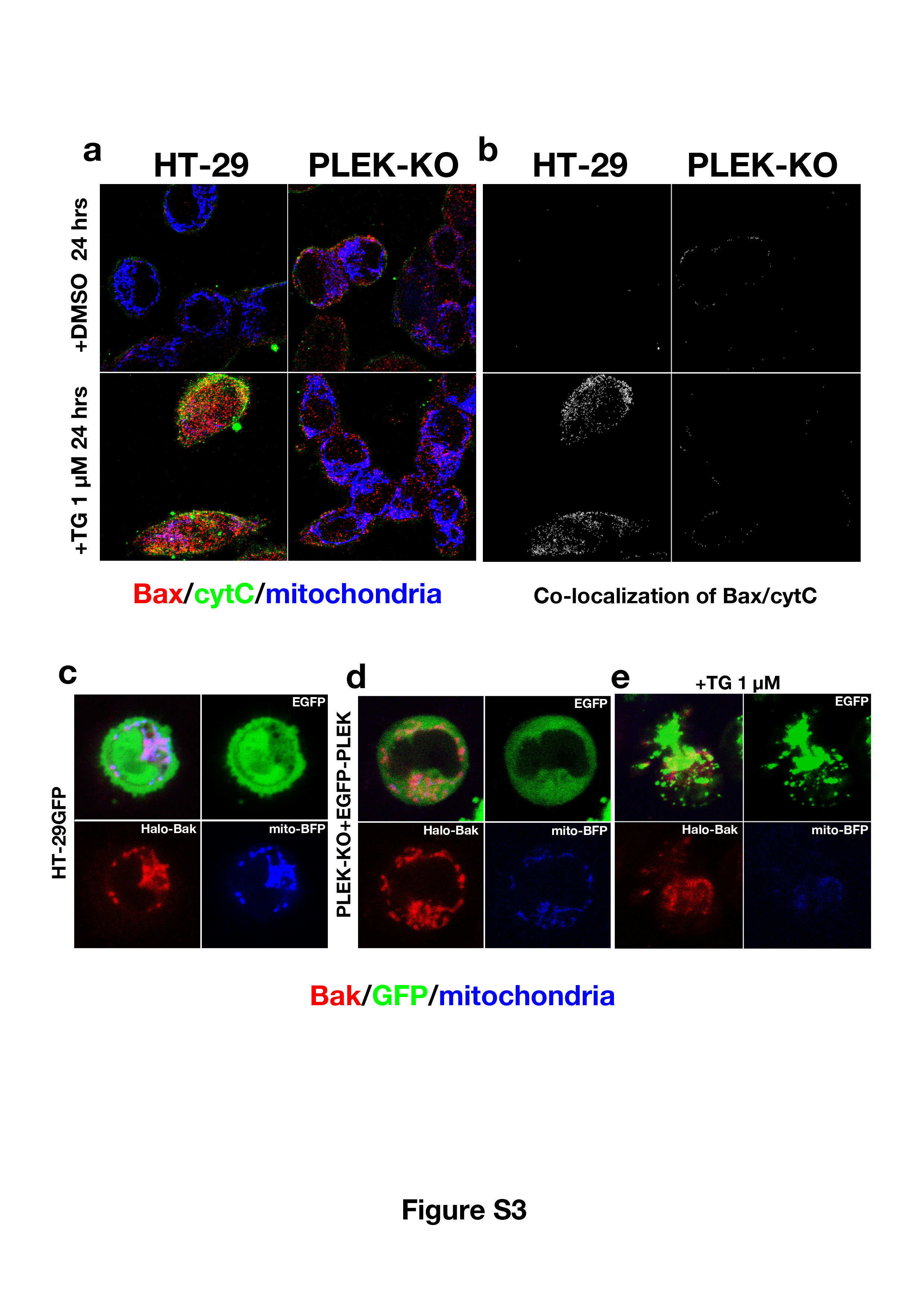

Supplement: Supplementary file 4 — Figure S3 [file 41420_2017_6_MOESM4_ESM.jpg]

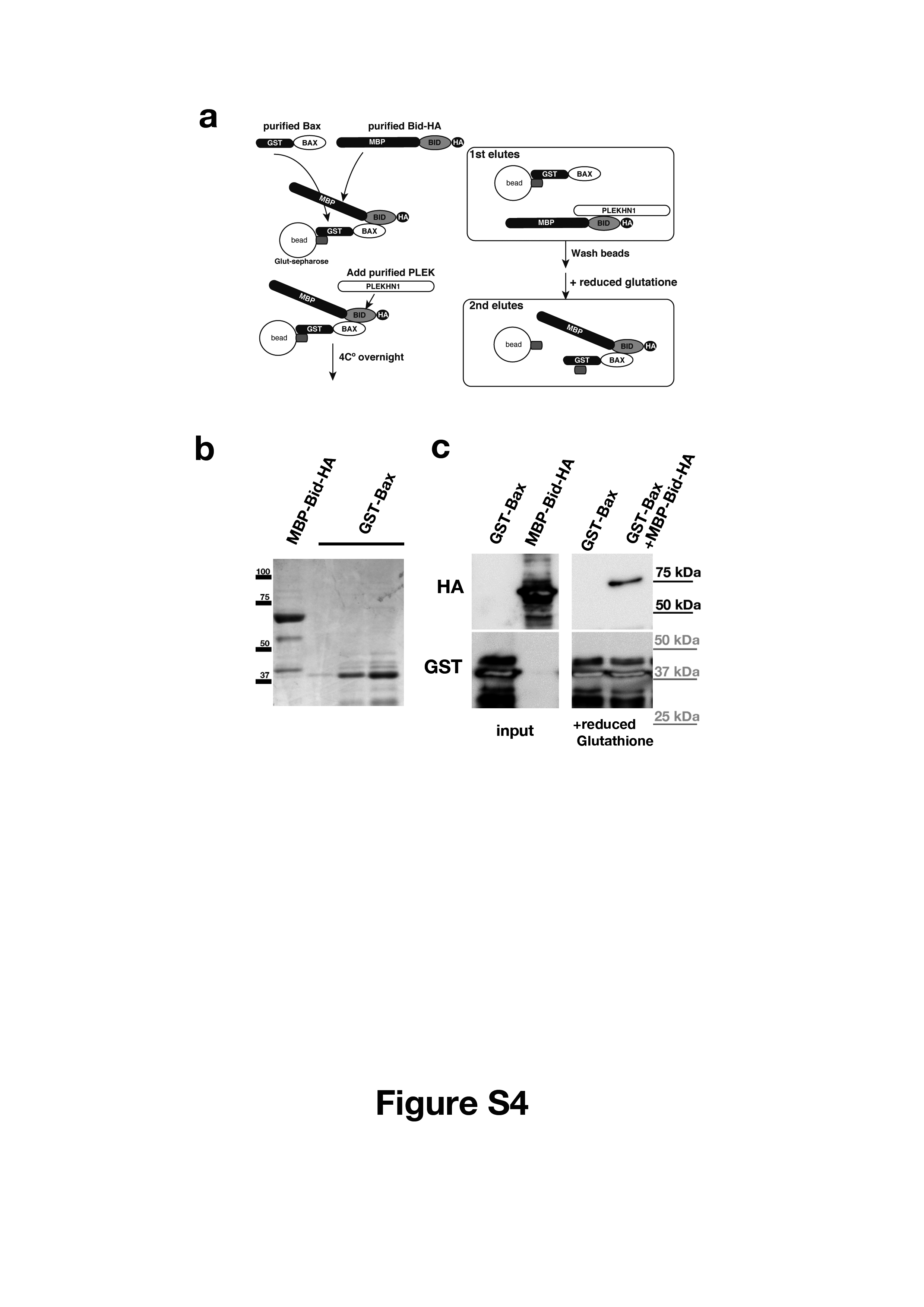

Supplement: Supplementary file 5 — Figure S4 [file 41420_2017_6_MOESM5_ESM.jpg]
